# How Long Halt Ye?

**Published:** 1920-12-04

**Authors:** 


### THE EDITOR'S LETTER-BOX.

**Correspondence on all subjects is invited, but we cannot in any way be responsible for the opinions expressed by our correspondents, who must give their name and address as a guarantee of good faith, but not necessarily for publication. Correspondents are reminded that brevity of style and conciseness of statement greatly facilitate early insertion.**

### "HOW LONG HALT YE?"

*To the Editor of* THE HOSPITAL.

DEAR SIR,—In reply to your leading article, I do not think that your criticism against me is entirely unfair.

You write that you wish I would show some sign of recognising that multitudinous pennies might save the situation, and that I have no ideas between personal appeals on the old lines and a complete abandonment of the old lines altogether. And you add that this attitude shows neither statesmanship nor good sense, but really a form of intellectual idleness. And you further state—this hurts me rather—that I rather like to startle the world with untempered announcements.

Tempting as it is to answer personal matters, I do not want to defend myself, because it really does not matter how much I lack statesmanship, good sense, and want of temper; but I do want to emphasise the fact that I have, for a whole year, with all the force at my command, and earnestness too, if you will believe it, been pressing the position of hospitals upon the Ministry of Health by numberless letters and by personal interviews and meetings with the leading officials, and I have told them at all these that unless help was forthcoming it would be impossible to go on, and Dr. Addison was told this by a deputation of hospital managers.

But I have only been met with sympathy and a *non possumus*. More than a year ago the Chancellor of the Exchequer wrote to me and said that the Government could not help. Then, when the Ministry of Health was appointed, I had more hopes. These hopes have vanished. And meanwhile, all hospitals, save a very few, have drifted into a worse and worse position. Those few are hospitals with large endowments, or with a special *clientèle* to whom they can successfully appeal, or, like Charing Cross, which Mr. Verity declares to be comfortably off.

But where a short time ago a "Close if you don't help us" appeal was made, it cannot be repeated. Even as regards the Charing Cross Hospital, I am told that but for a donation of £20,000 last year their accounts would not be very promising. All the hospital chairmen I meet tell me the same story—some more dismal than others.

If I am wrong, and more hospitals than I know of can go on, well, then, the committees to be appointed will prove this. I hope I am wrong. No one will own it more readily than I will.

I do recognise, *pace* your critic, that multitudinous pennies would save the hospitals, but they must be very multitudinous. The difficulty and expense of collecting these small sums would be very great, and of dividing them when obtained. And it would take a long time to organise a general collection of pence of sufficient magnitude to help the situation, which all the time would be getting more acute.

It seems to me that a rate would do this best, as the machinery is at hand. I think a rate fairer than general taxation, because rates touch those who use the hospitals as well as those who do not, whereas general taxation touches mainly those who do not use the hospitals, and those who do, escape. Moreover, I think a County Council a better judge of the wants of the county or district than the Government could be. I think, however, that State aid should be given to the great teaching hospitals, because they are doing national work as well as local.

It is no answer to tell me that this or that small hospital or country hospital has succeeded in getting multitudinous help from the working classes. In the first place, a comparatively small sum is needed, and in the next place you have in a county or district the *esprit de corps* for "our" hospital, which is lacking in London.

I quite agree with Mr. Coles that the insurance payments should be increased, and should carry the cost of institutional treatment, and I pressed this on Mr. Lloyd George when the Act was first introduced, and have had a scheme for this before the Minister of Health for many months.

But this will take time to carry, and, if it was carried, we must not forget that the insured people would then have a right to admission, and that the voluntary hospitals could not possibly meet the demands—and what about their dependants? Capt. Elliott, M.P., writes in the *Evening Standard* that to-day there are 28,000 patients who have been seen and examined, and are waiting for hospital treatment, but are denied solely on the ground of want of accommodation.

I think the "Approved Societies" ought to help hospitals generously. We save them enormous sums by lessening the number of weeks they have to pay sickness benefit to their members, and I think all hospitals ought to refuse to treat insured people till they do this. At the "London," and probably the experience of all hospitals is the same, 75 per cent. of our male and 25 per cent. of our women patients are insured people, and are members of these Approved Societies.

Last year we also had 12,000 patients referred to the out-patient department by panel doctors, very often for trivial ailments or injuries they could well have treated.

You accuse me of making an untempered announcement. I do not quite know what "untempered" means, but it is not an unconsidered announcement to say that with an expenditure before the London Hospital of £300,000 for 1921, and with a revenue (if the three funds give as before, and if the patients' payments amount to £20,000) of £100,000 or £150,000 at most, we could not carry on.

I was unwise to give January 1 as the day of closing, but I did not mean my statement to have the wide publicity it obtained, as the meeting where I made it was a small one attended only by medical men.

If, however, I have been instrumental in stopping the drift of doing nothing, and of saving the hospitals from getting into a still more impossible position, I shall rejoice at my announcement, even if it be untempered, and I will gladly be the scapegoat and bear all the kicks if the hospitals can get the ha'pence. We have waited too long for the "'something' to turn up."

Yours faithfully,

30th November, 1920. KNUTSFORD, Chairman.

